# Effects of Postpartum Fatigue and Depressive Cognitions on Life Satisfaction and Quality of Life in Arab Postpartum Women: The Intervening Role of Resourcefulness

**DOI:** 10.3390/nursrep11010009

**Published:** 2021-02-04

**Authors:** Hanan A. Badr, Jaclene A. Zauszniewski, Mary Quinn Griffin, Christopher J. Burant, Amy Przeworski, Wedad M. Almutairi, Fatmah H. Alsharif

**Affiliations:** 1 Department of Maternity and Child Nursing, Faculty of Nursing, King Abdulaziz University, Jeddah 21589, Saudi Arabia; 2 Bolton School of Nursing, Case Western Reserve University, Cleveland, OH 44106, USA; jaz@case.edu (J.A.Z.); mtq2@case.edu (M.Q.G.); cxb43@case.edu (C.J.B.); 3 Department of Psychological Sciences, College of Arts and Sciences, Case Western Reserve University, Cleveland, OH 44106, USA; amy.przeworski@case.edu; 4 Department of Medical Surgical Nursing, Faculty of Nursing, King Abdulaziz University, Jeddah 21589, Saudi Arabia; Walmutairi@kau.edu.sa (W.M.A.); falsharif@kau.edu.sa (F.H.A.)

**Keywords:** postpartum fatigue, depressive cognition, quality of life, life satisfaction, resourcefulness

## Abstract

The purpose of this study is to explore the relationships among postpartum fatigue (PPF), depressive cognitions, resourcefulness, quality of life, and life satisfaction in Arab postpartum mothers. A conceptual framework is used in this study based on the middle range theory of resourcefulness, which Zauszniewski developed in 2006. The study is a cross-sectional descriptive design with 123 postpartum women who had given birth within the past six months. used WhatsApp and Facebook for recruitment. developed the self-administered online survey in Qualtrics and collected data from 6 January 2017, to 6 February 2017. Correlation analysis is used to address the research aim and used the *P* value of 0.05 to determine the significance of the results. There were significant correlations among depressive cognitions and resourcefulness, life satisfaction, and quality of life; there were also significant correlations between PPF and life satisfaction, as well as among resourcefulness, quality of life, and life satisfaction. The results of this study emphasized the importance of assessing depressive symptoms and PPF in mothers early in the postpartum period. The results may contribute to designing future intervention studies aimed toward decreasing the risk of mothers with PPF developing more serious depressive symptoms.

## 1. Introduction

Postpartum fatigue (PPF) and depression are serious problems that affect women after childbirth [1]. In the United States, one in eight women experience depressive symptoms at this time [1], while around 14% of postpartum women in the eastern region of Saudi Arabia are affected by depression [2]. Furthermore, Alzahrani [3] found that 17.1% of primiparous mothers in Jeddah have such symptoms. In addition, approximately 63.8% of new mothers are affected by PPF, making this the most common problem for women during this period [4]. 

During the transitional period after birth, the new mother faces major changes in her life, social role, responsibilities, physiology, financial adjustments, and marital and interpersonal relationships. All these changes can lead to increased risk for developing postpartum depressive symptoms that may include PPF and depressive cognitions (DC). These symptoms influence the mother’s perception and judgement regarding her quality of life (QOL) and satisfaction with life (SWL) [5]. 

### Significance of This Study

The postpartum period is associated with major physiological, psychological, and social changes [5]. This leads to increased risk for PPF and DC. The latter are the initial predictors of the development, maintenance, and exacerbation of depressive somatic and affective symptoms [6], and depression is an ongoing problem that affects at least 15% of women after parturition [1]. Depression during the postpartum period has numerous adverse effects on the health status of both mother and newborn [7], negatively influences mother–newborn interactions [8], and it also negatively affects the mother’s social life and health [9].

PPF, specifically, affects more than 60% of mothers, making this the most common problem for women during the postpartum period [4]. Fatigue leads to a decreased capacity for physical and mental activity [10]. PPF is a dynamic phenomenon—it may stabilize or worsen as the postpartum period progresses [11]. It has been shown that PPF has a negative impact on the mother’s health and the newborn’s development. According to Kurth et al. [10], during the postpartum period, fatigue can decrease a mother’s concentration level and increase the incidence of PPD. As a result, it can heighten the risk of a mother doing harm to her newborn; can interfere with healthy mother–infant interaction; and can cause early weaning from breastfeeding, thus delaying infant development [12]. Despite PPF being a highly common ailment among new mothers, it garners very little attention from researchers and health care providers.

Additionally, PPF and depression have been found to negatively affect QOL and SWL in populations other than postpartum women [13,14]. Furthermore, several studies have shown that greater resourcefulness (RS) is effective in overcoming challenges and stressful situations [15,16]. Resourcefulness defined as the ability to perform daily tasks independently and to seek help from others when the person cannot function independently [17,18]. Researchers have found that RS protects individuals from different populations, such as older 1 adults, from being affected by the environmental and cognitive factors that could lead to depression and other psychological problems and affect SWL [19], but RS has not been previously studied in the postpartum female population.

Despite researchers having examined PPD in general and PPF from various perspectives, none have investigated the effect of PPF and DC on the mother’s QOL and SWL during the postpartum period. Moreover, no authors have explored the correlation of research variables among the population of postpartum women. Studying the effect of RS during the postpartum period is highly important because the mother faces many changes and challenges stemming from her new responsibilities and the changes in her life during this period [5]. RS skills are important during this period to help the mother adjust to her new responsibilities and roles.

**Figure 1:**
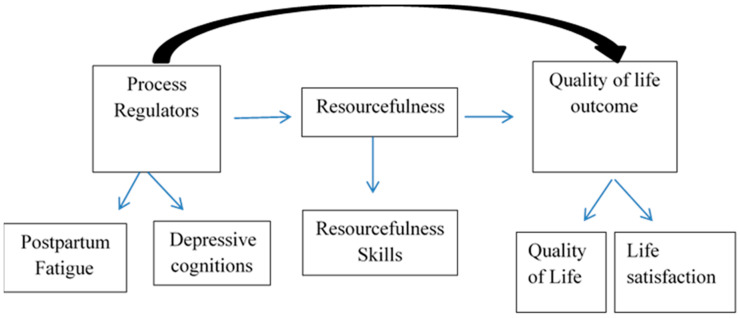
Research model for study of postpartum Arabic women.

The conceptual framework for this study was based on the middle-range theory of RS and QOL, first developed by Zauszniewski et al. in 2006 [18]. The framework is depicted in Figure 1. In this study, three constructs were drawn from the RS theory. The first construct is process regulators, which include intervening variables, such as cognition, affect, motivation, and perceptions [18]. Under this construct are two conceptual variables reflecting process regulators: PPF and DC. The variable for the construct of RS is RS skills. For the last construct, the mother’s QOL is reflected in measures of QOL and SWL during the postpartum period.

The aims of this study include the following: (a) To examine the relationships between process regulators (DC and PPF) and RS skills in mothers during the postpartum period, (b) to examine relationships between process regulators (DC and PPF) and QOL outcomes (QOL and SWL) in mothers during the postpartum period, and (c) to examine the association between RS skills and QOL outcomes (QOL and SWL) in mothers during the postpartum period. This study was focused on Arab postpartum women because most of these factors has not been studied before among this population.

## 2. Methods

### 2.1. Design and Sample

A cross-sectional descriptive correlational design was used to examine the relationships among PPF, DC, RS, QOL, and SWL. The sample of the study was a convenience sample of postpartum women who had given birth at least 2 weeks and not more than 6 months previously. We used a convenience sample because this is the first study to constitute an attempt to find correlations among the study variables pairings. In addition, the study design was descriptive, and there was no database from which study participants could be randomly selected.

The sample size was determined based on correlational power analyses that should be conducted as two-tailed tests, an α of 0.05, power of 0.80, and ES of 0.15 yielded a need for 85 subjects to provide a complete data set. To compensate for missing data or attrition of the participants, the study needed to include 120 women.

### 2.2. Subjects (Inclusion and Exclusion Criteria)

The inclusion criteria for the study were as follows: (1) The mother must be able to speak Arabic; (2) the mother should have her newborn with her at home; (3) the mother should be 18 years of age or older; and (4) the mother should have given birth at least 2 weeks and not more than 6 months before participation. Data on mothers during the first 6 months of the postpartum period were included because this period is the most difficult time and because most previous authors had collected data for this period [12,19] In addition, only mothers at least 2 weeks postpartum were included because maternity blues and psychosis usually manifest in the first 2 weeks. By excluding this time period, we avoided confusion between participants experiencing maternity blues or psychosis and participants experiencing DC symptoms [20].

The exclusion criteria for the study were (a) mothers with newborns who were dependent on technology after discharge. According to Toly et al. [21], mothers of children who were dependent on technology such as (mechanical ventilation, oxygen, or intervenors nutrition or medications) experienced a higher level of depressive symptoms; (b) mothers who experienced a multiple birth; and (c) mothers who were pregnant at the time of the study. These mothers were excluded because they were expected to have both a high level of fatigue and a high risk for developing depression [7,22].

### 2.3. Recruitment and Data Collection

After institutional review board approval was obtained, the participants were recruited through Arab groups on Facebook and WhatsApp. For data collection, a self-administered online survey developed using Qualtrics was administered. In addition, the participants were instructed to sign a waiver of written consent before starting the survey. If the participants were willing to take part, they were asked to answer the screening questions to assess their eligibility. If found eligible, they were directed to the consent form, where they were asked to check a box that served as their electronic signature.

### 2.4. Measurements

***Postpartum fatigue.*** The Multidimensional Assessment Fatigue scale (MAF) was used to measure PPF. The MAF scale is a self-reported questionnaire composed of 16 items divided into 4 dimensions. These dimensions are (a) severity of fatigue; (b) distress; (c) impact of fatigue on activities of daily living (cooking, doing household chores, bathing, dressing, working, participating in social life, sexual activity, leisure and recreation, shopping, walking, and exercising); and (d) frequency of fatigue over the previous week. The level of measurement of this scale is a ratio, and the resulting score ranges from 1 (*no fatigue*) to 50 (*severe fatigue*) [23]. Higher scores mean greater fatigue, distress, and interference with activities of daily living [24,25]

The MAF scale was developed in the English language by Belza in 1991 [26]. However, for this study we used an Arabic version of the scale, developed using a translation and back-translation process, and the psychometric evaluation performed by Bahouq et al. [24]. The Arabic version of the MAF scale demonstrated an acceptable Cronbach’s alpha at 0.70 and good validity and sensitivity [24]. For the current study, the Cronbach’s alpha for MAF was measured at 0.84, which is considered good reliability.

***Depressive cognitions.*** DC were measured by the Depressive Cognitions Scale (DCS), which was developed by Zauszniewski and translated into Arabic by Bekhet [27]. The DCS assesses 8 depressive thoughts: Hopelessness, helplessness, powerlessness, purposelessness, worthlessness, loneliness, emptiness, and meaninglessness. All items are phrased positively and need to be reverse coded before analysis. The total score ranges from 0–40, and the higher the score after reverse coding of the 8 items, the greater the level of DC [27]. The cutoff for the DCS is 7, which means patients with scores greater than 7 have a higher risk for developing clinical depression [28]. The DCS-Arabic has an acceptable internal consistency (α = 0.78) and has demonstrated good construct validity [29]. For the current study, the Cronbach’s alpha was 0.75, which is considered acceptable reliability.

***Resourcefulness.*** In this study the Resourcefulness Scale (RS) was used to measure the RS concept [18]. The scoring method for the RS is a 6-point Likert scale ranging from 0 (*not at all like me*) to 5 (*very much like me*). The total score ranges from 0–140. Higher scores mean higher personal and social resourcefulness [18,29]. The Arabic version of RS showed acceptable reliability (0.81) and good validity [30], and in the current study, the reliability was acceptable (α = 0.78).

***Quality of life.*** A single-item, global-QOL question was used to measure QOL. The responses ranged from 1 (*poor*) to 5 (*excellent*) [31]. The single-item, global-QOL question was compared with the standard health measure to assess the construct validity of the question. The researcher found that the single-item, global-QOL question had good discriminant validity with the physical capacity and psychological distress scales [31]. This item was the most suitable for our study because it assessed both health-related and non-health-related aspects of QOL [32].

***Life satisfaction.*** In this study, SWL was measured by the Satisfaction with Life Scale (SWLS). This scale measures the judgmental component of subjective well-being [33]. The scoring method is based on a Likert scale ranging from 1 (*strongly disagree*) to 7 (*strongly agree*). The range of possible scores is 5 (*minimal satisfaction with life*) to 35 (*very high satisfaction*) [33,34]. The Arabic version of the SWLS was translated by Abdallah [34] and shows acceptable reliability (α = 0.79) and good validity. Reliability for the current study was 0.86, which is considered acceptable.

### 2.5. Data Analysis

The data were automatically entered from Qualtrics to SPSS. Data screening was performed by running descriptive statistics and frequencies for all variables to check for missing data, outliers, and unusual scores that could cause problems during the analysis. The statistical analysis used for the research questions was correlation, which was used because it helped determine the relationships among the variable’s pairs. In addition, it helped define the direction of association between the variables (positive or negative) as well as the effect size (the magnitude) [35]. The *p* value that was used to determine the significance of the results was (0.05).

## 3. Results

The data were collected between January and February 2017, and 123 Arab postpartum women completed the survey. All participants included in this analysis were married women, and 70.7% had experienced a vaginal delivery. The other demographic characteristics were displayed in Table 1.

### 3.1. Descriptive Statistics for the Composite Scale

The results of all the scales showed variance in the data, and all data were normally distributed, based on the skewness and kurtosis of the total score of the scales. All the information for the mean, standard deviation, and minimum and maximum values is displayed in Table 1.

The DC mean was 5.69, which is considered to be a low score based on the published cutoff score of 7 on the DCS [28]. The results showed that 69.1% of the participants scored between 0 and 6, 23.7% scored between 7 and 13, and 7.2% scored between 14 and 26, which indicates that 30.8% of the participants were at higher risk for developing clinical depression [28]. The postpartum fatigue mean was 8.5, which was considered a moderate fatigue level. Results also showed that 9% of the participants had a low fatigue level, 54.9% had a moderate fatigue level, and 36.1% had a high fatigue level.

The RS mean was 93.2, which was considered a moderate level based on the RS score range mentioned by Zauszniewski et al. [36]. In this study, 2.4% had low RS, 10.6% had somewhat low RS, 47.2% had moderate RS, 35.7% had somewhat high RS, and 4.1% had high RS.

In addition, the SWL mean was 25.1, which was considered a high satisfaction level. The QOL item had 5 responses, and the QOL mean was 3.45, which was considered a very good QOL. The percentages for each response were 20.8% *excellent*, 33.6% *very good*, 20% *good*, 21.6% *fair*, and 4% *poor*. The other descriptive statistics for all the scales are shown in Table 2.

### 3.2. Correlations among the Variables

The results of the analysis showed that the strongest significant correlations were between DC and RS, PPF and SWL, and RS and QOL and that the RS and SWL correlation was also significant. Finally, there were no significant correlations between PPF and RS or PPF and QOL (refer to Table 3).

## 4. Discussion

In this study, 123 postpartum women completed a survey relating to PPF and DC. The percentage of participants between 26 and 30 years old was 41.5%, which is consistent with previous research on postpartum women [18,36]. In addition, 70.7% of the participants had had vaginal births, and 29.3% had had cesarean sections. These results are similar to the findings from Taylor and Johnson’s study [18], in which 78% of the participants had had vaginal births and 22% had had cesarean sections.

This study is significant because it is one of the first to offer an examination of the correlations among the previously mentioned factors in Arab women, specifically within the context of RS theory. A significant correlation between DC and RS was found, which is consistent with the findings of Zauszniewski et al. and Zauszniewski et al. [37,38], although these two studies were not conducted with postpartum women. Unfortunately, the correlation between DC and RS in postpartum women had not previously been studied, and neither had that between PPF and RS.

DC was significantly correlated with QOL and SWL, which is consistent with RS theory and the previous study by Zauszniewski et al. [38]. However, the participants of said study were female relatives of mentally ill adult patients. Our results were also consistent with other studies whose authors investigated the correlation between depression or depressive symptoms and QOL and SWL [39]. Wells et al. [39] found that patients with depressive symptoms reported limitations in QOL. Moreover, Strine et al. [13]. found similar results indicating that depressed patients suffered impairment in their health-related QOL and that they were not satisfied with their lives [40]. Underhill et al. [40] noted that survivors of traumatic brain injuries who were diagnosed with depression also reported lower SWL.

Two studies involving postpartum women were found in the literature, and their results are consistent with our findings. However, the focus of these studies was PPD, not DC, which is known to precede the development of clinical depression [41]. Sadat et al. [42] found medium negative correlations between PPD and QOL at 2 and 4 months, and Boyce et al. [43] found that PPD negatively affected the 5 dimensions of QOL.

In terms of PPF, a limited number of studies have been focused on the effect of PPF on the mother’s QOL [44,45]. However, some authors have investigated the correlation between fatigue in general and QOL among several populations, such as cancer patients [14] and adults with chronic diseases [46], but none have specifically focused on the association of fatigue with SWL.

In this study, PPF had a significant negative correlation with SWL, but its correlation with QOL was not significant. Our study findings were not consistent with the findings of previous researchers. Gupta et al. [14] found that fatigue levels among cancer patients were negatively and strongly associated with QOL. In addition, Michielsen et al. [47] conducted a study among sarcoidosis patients and found that fatigue was negatively associated with all domains of QOL. Elsewhere, another study was performed among adults who had chronic insomnia, and the researchers found the same strong correlation between fatigue and QOL [46].

The possible reasons for finding inconsistent results include: (1) Studies of the concepts were among different populations, and (2) researchers used different scales to measure the concepts. In the three previously mentioned studies, the researchers used scales that differed from the scale used in this study to measure QOL. In this study, the single-item global QOL question was used, but Fortier-Brochu et al. [46] used SF−36, Michielsen et al. [47] used the WHOQOL-100, and Gupta et al. [14] used the Ferrans and Powers QOL Index. The single-item global QOL question measured the participants’ perceptions of their QOL in general. However, the other QOL scales consist of various domains. For example, the SF-36 consists of 8 domains: General health, mental health, physical functioning, social functioning, bodily pain, vitality, restriction of usual activities due to physical problems, and emotional problems [46]. The WHOQOL-100 measures 6 domains: physical health, psychological health, level of independence, social relationships, environment, and spirituality [46]. The Ferrans and Powers QOL Index has 4 major domains: health and physical, social and economic, psychological and spiritual, and family [14]. Also, As mentioned previously, all studies [44,45,46,47] focused on populations with chronic diseases; however, 70% of the mothers in our sample did not have any chronic diseases, and 78% had at least one person to provide support for them. All these factors could explain the lack of correlation between PPF and QOL.

Furthermore, as expected, RS had a significant positive correlation with QOL and SWL. The study results were consistent with RS theory and previous studies whose authors investigated the correlation between RS and QOL and between RS and SWL. Examples of previous studies to feature investigations of the correlation between RS and QOL are Huang et al. [48], who studied women with breast cancer, and Zauszniewski et al. [38], who studied chronically ill patients. In addition, the correlation between RS and SWL was consistent with the results of two Zauszniewski [49,50] studies, which were focused on elderly participants.

### Strengths and Limitations of the Study

This study has several strengths and some limitations. One of its strengths is that it is one of the first studies to offer an examination of these types of variables in Arab women, and the sample size is fairly large. In addition, a previously tested theoretical framework was used to help design the study, and all scales used were valid and reliable. In addition to these strengths, a few points may be considered as both strengths and limitations at the same time. Using online data collection made it easy for the mothers to complete the survey when they had the time, but it also increased the probability of incomplete surveys and missing data. Using social media for recruitment to reach a large number of participants was a strength, but it was also a limitation in terms of missing potential participants who do not use such media.

Other limitations of this study included the use of a convenience sample and the unvaried demographic characteristics of the participants. All enrollees were married and highly educated, which limits the generalizability of the results to the participants enrolled in this study. Finally, the descriptive, cross-sectional design is another limitation because the data were collected at the same time, and thus, we could not measure changes in the variables over time.

## 5. Conclusions

Despite its handful of limitations, the results of this study indicate that DC have significant correlations with RS and QOL outcomes. Postpartum fatigue correlated only with SWL, and RS correlated with both QOL and SWL, supporting the value of RS theory for this population. In addition, these findings emphasize the importance of assessing depressive symptoms and PPF in mothers during the first weeks of the postpartum period to help them adjust to their new responsibilities and roles and to safeguard their physical and psychological well-being.

The results also suggest that to improve postpartum women’s psychological and physiological health and QOL, policymakers in Arabic countries should mandate the assessment of the mother’s depressive symptoms and fatigue level during the first six months of the postpartum period. This assessment should be conducted before discharge from the hospital, at three months postpartum, and at six months postpartum.

Furthermore, health policymakers should take into account the importance of assessing the QOL of postpartum women because this will affect not only them but also their newborn and their entire family. Hospitals should adopt a policy to provide postpartum women with all the available resources, support, and education they can concerning the mother’s health during the postpartum period to help her adapt to her new responsibilities.

Moreover, nurses should provide information about educational programs prior to delivery to improve women’s QOL during the postpartum period by educating them and their partners on the expected changes and responsibilities at this time. In addition, nurses should educate mothers on some self-management techniques that will help them adapt to their new responsibilities, decrease their stress levels, and help them maintain their physical and psychological health. Finally, nurses should try to personalize postpartum education based on the individual mother’s needs to avoid overwhelming her with information that might not be related to her circumstances.

Several suggestions can be made for future research, which include replicating the study in a hospital setting and assessing mothers’ DC and PPF levels over time to determine whether they affect their SWL and QOL. Moreover, one of the possible recommended studies is to investigate whether there is any correlation between the process regulator variables (PPF and DC) and between the QOL outcome variables (QOL and SWL). The latter may help in determining whether these two concepts are different or whether they overlap in postpartum women, which will help define and measure these two variables in future research.

In addition, conducting mixed-methods research is suggested because it will provide a better understanding of the perceptions, life experiences, and cultural viewpoints of postpartum women, as well as of the quantitative data collected from the mothers. Such research will enrich our knowledge of the postpartum phenomenon.

## Figures and Tables

**Table 1:** Demographic characteristics.

Demographics		Percent %		
Age	18–25 years	20.3		
	26–30 years	41.5		
	31–35 years	26		
	36–40 years	9.8		
	More than 40 years	2.4		
Employment Status	Full time employee	35.8		
	Part time employee	5.7		
	Working from home	2.4		
	Unemployed	56.1		
Education	Elementary school	0.8		
	Middle school	0		
	High school	7.3		
	2 years of college or technical school	5.7		
	Bachelor degree	58.5		
	Master degree	22		
	Doctorate degree	5.7		
Presence of Social support	Yes	78		
	No	22		
	Minimum	Maximum	Mean	SD
Number of pregnancies	1	10	2.73	1.7
Number of children	1	7	2.3	1.4

**Table 2:** Descriptive Statistics for the Composite Scores of the Scales.

Variable	M	SD	Skewness	Kurtosis	Min	Max
Depressive cognitions (DC)	5.69	4.72	1.62	3.45	0	26
Postpartum fatigue (PPF)	28.5	9.62	−0.496	0.144	2	47
Resourcefulness (RS)	93.2	15.88	−0.36	0.283	43	129
Satisfaction with life (SWL)	25.1	6.37	−0.697	−0.232	7	35
Quality of life (QOL)	3.46	1.16	−0.30	−0.95	1	5

**Table 3:** Correlations Between the Variables.

Correlations	Results	Comments
PPF→ RS	*β* = 0.059, *p* = 0.52	Not Significant
DC→ RS	*β* = (−0.37), *p* < 0.0001	Medium Negative Correlation
PPF→ QOL	*β* = −0.097, *p* = 0.29	Not Significant
PPF→SWL	*β* =(−0.24), *p* = 0.007	Low Negative Correlation
DC→QOL	*β* = (−0.48), *p* < 0.001	Medium Negative Correlation
DC→SWL	*β* = (−0.43), *p* < 0.001	Medium Negative Correlation
RS→QOL	*β* = 0.27, *p* = 0.001	Low Positive Correlation
RS→ SWL	*β* = 0.21, *p* = 0.017	Low Positive Correlation

## Data Availability

Not applicable.
